# Cognitive functioning following stabilisation from first episode mania

**DOI:** 10.1186/s40345-017-0108-2

**Published:** 2017-12-18

**Authors:** Rothanthi Daglas, Kelly Allott, Murat Yücel, Lisa P. Henry, Craig A. Macneil, Melissa K. Hasty, Michael Berk, Sue M. Cotton

**Affiliations:** 1 Orygen, The National Centre of Excellence in Youth Mental Health, 35 Poplar Road, Parkville, VIC 3052 Australia; 2 Centre for Youth Mental Health, University of Melbourne, 35 Poplar Road, Parkville, VIC 3052 Australia; 3 Brain and Mental Health Laboratory, School of Psychological Sciences & Monash Biomedical Imaging Facility, Monash University, Clayton, Australia; 4 Orygen Youth Health-Clinical Program, 35 Poplar Road, Parkville, 3052 Australia; 5 IMPACT Strategic Research Centre, School of Medicine, Deakin University, PO Box 281, Geelong, 3220 Australia; 6 Barwon Health and the Geelong Clinic, Swanston Centre, PO Box 281, Geelong, VIC 3220 Australia; 7 Florey Institute for Neuroscience and Mental Health, Kenneth Myer Building, Royal Parade, Parkville, Australia

**Keywords:** Bipolar disorder, Cognition, Depression, Manic, Remission

## Abstract

**Background:**

The purpose of this study was to examine cognitive functioning in people following first-episode mania relative to a demographically similar healthy control group.

**Methods:**

Forty-one patients, who had recently stabilised from a first manic episode, and twenty-one healthy controls, were compared in an extensive cognitive assessment.

**Results:**

First-episode mania participants had significantly lower Full-Scale IQ (FSIQ) relative to healthy controls; however, this finding could be driven by premorbid differences in intellectual functioning. There were no significant differences between groups in Verbal IQ (VIQ) and Performance IQ (PIQ). First-episode mania participants performed significantly poorer than healthy controls in processing speed, verbal learning and memory, working memory, and cognitive flexibility with medium-to-large effects. There were no group differences in other measures of cognition.

**Conclusions:**

Participants following first-episode mania have poorer global intelligence than healthy controls, and have cognitive difficulties in some, but not all areas of cognitive functioning. This highlights the importance of early intervention and cognitive assessment in the early course of the disorder.

## Background

Cognitive impairments during euthymia in people in all stages of bipolar disorder have been reported by several meta-analyses, with findings of medium to large effect in processing speed, verbal learning and memory, non-verbal memory, working memory, verbal fluency, sustained attention and executive functions (Arts et al. 2008; Bora et al. 2009; Bourne et al. 2013; Mann-Wrobel et al. 2011). There has been limited research conducted on cognitive functioning during the early stages of the illness, thus the timing and onset of cognitive change remains unclear.

Recently, two meta-analyses on adults with first episode bipolar disorder (including any polarity of illness) have identified impairments in most cognitive domains relative to healthy control (HC) participants (Bora and Pantelis 2015; Lee et al. 2014). However, a recent systematic review on first episode mania (FEM) that included adolescents as well as adult samples, revealed discrepancies between study findings across most cognitive domains, apart from a consistently reported deficit in working memory during remission, and that verbal fluency and non-verbal memory remained intact (Daglas et al. 2015). The literature regarding intelligence in FEM has revealed that patients perform worse than HCs in global IQ (Elshahawi et al. 2011; López-Jaramillo et al. 2010), and on measures of non-verbal intelligence (Hellvin et al. 2012; Torres et al. 2010); however, the studies in FEM (included in the systematic review) had not controlled for the differences observed between FEM and HC groups in IQ, which may have contributed to the inconsistencies between study findings (Daglas et al. 2015). This is in contrast to a meta-analysis that reported preserved current IQ in people with established bipolar disorder (Bora et al. 2009). Moreover, there appears to be a bi-modal pattern regarding school performance in asymptomatic adolescents (aged 15–16 years) who later develop bipolar disorder. Those with the highest school grades displayed a nearly fourfold increased risk of developing the disorder by age 31, whilst those with the lowest school performance were found to have close to a twofold increased risk (MacCabe et al. 2010). Predictors of lower intellectual functioning in individuals with bipolar disorder include an earlier age of onset, greater number of prior episodes and hospitalisations (Denicoff et al. 1999).

Although cognitive deficits have been identified during remission in bipolar disorder, the effects of intelligence, residual symptoms, medication use, number of hospitalisations and number of past episodes may impact upon the findings (Bourne et al. 2013; Donaldson et al. 2003; Nehra et al. 2006; Thompson et al. 2005; Torres et al. 2010). The first diagnostic episode for bipolar I disorder enables the assessment of cognitive functioning prior to the effects of covariates such as prolonged medication use and recurrent manic episodes. Most of the studies conducted to date have included adult samples with FEM (Daglas et al. 2015) and have not considered that the incidence of FEM is greatest between 16 and 30 years (Kennedy et al. 2005), and that there may be developmental differences in cognition between younger and older populations.

Thus, the aim of the current study was to examine cognitive functioning of young people (15–25 years) following FEM relative to a demographically similar HC group. The cognitive domains considered were processing speed, attention, sustained attention, verbal learning and memory, non-verbal learning and memory, working memory, verbal fluency and executive functions. Due to diagnostic instability in the early phases of the illness, and that an accurate diagnosis is on average delayed for 7.5 years in people with bipolar disorder, any presentation of FEM will be considered in this study (Ghaemi et al. 1999; Schimmelmann et al. 2005). It was hypothesised that FEM participants would perform significantly worse than HCs in processing speed, attention, sustained attention, verbal learning and memory, working memory and executive functions; and that the groups would not significantly differ in verbal fluency and non-verbal memory. Furthermore, FEM participants were expected to perform more poorly than HCs in full-scale IQ (FSIQ) and performance IQ (PIQ), but that the groups would have similar verbal IQ (VIQ).

## Methods

### Design

This study involved secondary analysis of baseline data from a single-blinded Randomised Control Trial (RCT) conducted at Orygen, The National Centre of Excellence in Youth Mental Health, Melbourne, Australia. The focus of the trial was on the effectiveness of quetiapine fumarate compared to lithium carbonate monotherapy for the maintenance treatment of FEM over a 12-month period. This trial was registered with the Australian and New Zealand Clinical Trials Registry ACTRN12607000639426. Neuropsychological data were also collected on a separate HC group that was not part of the RCT, using the same time-points and measures.

### Sample and setting

The FEM patients were recruited between 2006 and 2013 from outpatient clinics of OYH and Monash Health, located within the Western, North Western and South Eastern suburbs of Melbourne, respectively. To satisfy inclusion criteria for the RCT, the FEM patients were required to have: (1) clinically stabilised from a first treated manic episode on a combination of quetiapine and lithium for a least 1 month prior to randomisation (stabilisation of mental state was based on clinical judgment by the treating clinicians on the basis of a global clinical assessment); (2) met Diagnostic and Statistical Manual of Mental Disorders-Fourth Edition-Text Revision (DSM-IV-TR; APA, 2000) criteria for bipolar I disorder, schizoaffective disorder-bipolar type, or a substance-induced mood disorder; (3) scored a minimum of 20 on the Young Mania Rating Scale (YMRS) during the first manic episode; and (4) been aged 15–25 years at the time of recruitment.

FEM patients were excluded if they had a clinically relevant systemic medical disorder, biochemical or haematological abnormalities or unstable diabetes mellitus, were pregnant or lactating, had a sensitivity or allergy to components of lithium or quetiapine, were non-fluent in English, had a history of epilepsy, were at immediate risk of harm to self or others, or had an organic mental disease including intellectual disability (FSIQ < 70). The use of potent cytochrome P450 inhibitors and inducers was also prohibited during the study.

HCs were matched as closely as possible to the FEM group in age, sex and premorbid intelligence, and were recruited from similar regions of Melbourne through advertisements in a freely distributed newspaper at inner-city metro stations of Melbourne, the Orygen website, and by word of mouth. Due to recruitment feasibility, FEM and HC participants were recruited at a ratio of 2:1. Individuals interested in participating contacted the researchers and were given a detailed description of the study. Prior to providing informed consent, the HCs were assessed for current or past mental health disorders with the screening tool of the Structured Clinical Interview (Patient Edition) for DSM-IV-TR. HCs were excluded if they had a history of, or current mental health disorder, substance abuse or dependence, an FSIQ < 70, or were not 15–25 years of age.

### Measures

#### Neuropsychological measures

One assessor (RD) was trained in standardised neuropsychological testing and clinical assessment of this clinical population and conducted all assessment components of the study. The neuropsychological battery was administered in the same order for all participants and consisted of psychometrically robust, valid and standardised tests. Cognitive functions that were assessed included intelligence, processing speed, attention span, sustained attention, verbal learning and memory, non-verbal learning and memory, working memory, verbal fluency and executive functions.

#### Intelligence

To measure estimated premorbid intelligence, the Wechsler Test of Adult Reading (WTAR) was used (The Psychological Corporation 2001). As a measure of current intelligence, the Wechsler Abbreviated Scale of Intelligence (WASI) was utilised (The Psychological Corporation 1999). From the WASI, FSIQ, VIQ and PIQ scores were derived.

#### Processing speed

Four tests of processing speed were administered, including Trail Making Test—part A (TMT-A) (Reitan 1955), WAIS-III Digit Symbol Coding (Wechsler 1997), the computerised Go/No-Go test to assess the reaction time for go responses (see Takagi et al. 2011), and the Cogstate_TM_ Detection task (see Hammers et al. 2011, 2012).

#### Attention

Attention span was assessed with WAIS-III Digit Span-forward (Wechsler 1997). The computerised Stroop task for congruent responses, Go/No-Go test for missed go responses (see Takagi et al. 2011), and Cogstate_TM_ Identification task were used as measures of focused attention (see Hammers et al. 2011, 2012).

#### Sustained attention

A shorter version of the original Attention Network Test (ANT) was used to measure sustained attention (Fan et al. 2005).

#### Verbal learning and memory

The Rey auditory verbal learning test (RAVLT) was administered to test verbal learning and memory (Lezak 1983; Rey 1958). The correct recall of words in trial 1 was used as a test of immediate memory, and the total correct recall of the same list of words in five consecutive trials was used to measure verbal learning. Delayed verbal recall was measured by the recall of the same list of words after a 20-min interval.

#### Non-verbal learning and memory

The computerised Cogstate_TM_ One-card leaning task (OCL) (see Hammers et al. 2011, 2012) and the Cogstate_TM_ Groton Maze Learning Test (GMLT)—delayed recall were used as measures of non-verbal learning and memory (see Snyder et al. 2005).

#### Working memory

The WAIS-III Digit Span-backward was used to measure working memory capacity (Wechsler 1997).

#### Verbal fluency

The Controlled Oral Word Association Test was used to measure semantic (animal category) and phonemic (FAS) verbal fluency (see Mitrushina et al. 2005).

#### Executive functioning

The Trail Making Test—part B (TMT-B) was utilised to measure cognitive flexibility (Reitan 1955). To assess inhibitory control, computerised versions of the Stroop and Go/No-Go tasks were used (see Takagi et al. 2011). The Cogstate_TM_ GMLT was used as a test of spatial problem solving (see Snyder et al. 2005).

#### Clinical measures

The clinical scales used to assess psychiatric symptomatology included: the YMRS (Young et al. 1978); the Montgomery–Åsberg Depression Rating Scale (MADRS) (Montgomery and Asberg 1979); the Brief Psychiatric Rating Scale (BPRS), total scores and positive psychotic scores (including the 4 subscales: unusual thought content, hallucinations, suspiciousness and conceptual disorganisation) (Overall and Gorham 1962); and the Clinical Global Impression scale—modified for bipolar disorder (CGI-BP) (Spearing et al. 1997).

### Procedure

The trial adhered to Good Clinical Practice guidelines, and was approved by the Human Research Ethics Committees of Melbourne Health (HREC 2006.644) and Monash Health (06138B). All participants or legal guardians (on behalf of participants under 18 years of age) provided voluntary informed consent. Patients who were treated with a combination of quetiapine and lithium for their first acute episode of mania were referred to the study by the treating psychiatrist or case manager. Once patients had clinically stabilised and were transferred to outpatient care, they were provided with a full description of the study. Cognitive and clinical assessments occurred once the patients had stabilised and had commenced monotherapy. The clinical assessment was conducted on the same day as the cognitive assessment for most FEM participants (41%). Thirty-one percent of the FEM participants had the clinical assessment within the first week, 15% within the 2-weeks and 13% over 2-weeks of cognitive testing. The time-point for the structured clinical interview for DSM-IV-TR was within 2–4 weeks of the cognitive testing.

### Data analysis

All statistical analyses were conducted using IBM^®^ SPSS^®^ Statistics Version 22.0. Descriptive statistics were calculated for demographic variables, and illness characteristics. Independent samples *t* test and Chi-square (*χ* ^*2*^) analyses were performed to assess for between-group differences on demographic variables. Several outliers were identified by box plots, and skewness and kurtosis values revealed that the cognitive data were differentially distributed within the two groups. Therefore, non-parametric Mann–Whitney *U* tests were utilised to compare groups for each cognitive measure. To control for the effects of multiple comparisons, family-wise error adjustments were made per cognitive domain (*α* = .05/number of cognitive measures per domain). To determine absolute values for effect size (*r*), *z* scores were divided by the square root of *N* (*r* = *Z*/√*N*), as used for non-parametric tests (Fritz et al. 2012). According to Cohen’s (1998) guidelines for *r*, a small effect size is 0.1, medium effect size is 0.3 and a large effect size is 0.5 (Coolican 2009). The relationship between clinical symptom rating (YMRS, MADRS, BPRS and BPRS—positive psychotic scores) and cognitive functioning was assessed using Pearson’s correlation on any measure that showed a significant difference between FEM participants and HCs following family-wise adjustment.

## Results

The cohort included sixty-one patients who had recently stabilised from their first treated manic episode. Of the 61 FEM patients, 7 were excluded due to not adhering to the randomised medication allocation, 2 were deemed too unwell to participate by their treating psychiatrist, 5 relapsed prior to the first assessment, and 6 withdrew consent or disengaged from the service. In total, 41 FEM participants and 21 HCs were included in the study.

### Sample characteristics

Demographic and illness characteristics are presented in Table 1. The FEM and HC groups did not differ significantly in age, sex and premorbid intelligence. However, the difference observed between groups in premorbid intelligence was of moderate effect (*d* = 0.57). On average the HC group had spent more years in education than the FEM group. The largest percentage of FEM patients had a diagnosis of bipolar I disorder (85%), 10% had a substance-induced mood episode and 5% were diagnosed with schizoaffective disorder-bipolar type. All FEM participants had experienced a manic or mixed episode with psychotic features.

**Table 1:** Participant demographics and illness characteristics

Demographics variables	FEM (*n* = 41)	HC (*n* = 21)	Statistics	*p* value
Age, M (SD)	21.32 (2.26)	21.14 (2.54)	*t* test	0.784
Sex, male, n (%)	32 (78)	11 (52.38)	*χ* ^*2* c^	0.074
Premorbid intelligence^a^				
Based on WTAR UK norms, M (SD)	94.9 (13.81)	101.70 (9.65)	*t* test	0.054
Education level				
Less than 12 years, n (%)	16 (39)	2 (7)	*χ* ^*2*^	0.032
12 years, n (%)	7 (17)	3 (14)		
More than 12 years, n (%)	18 (44)	16 (76)		
Diagnosis				
Bipolar I disorder^b^, n (%)	35 (85)			
Substance-induced mood disorder, n (%)	4 (10)			
Schizoaffective disorder- bipolar type, n (%)	2 (5)			
Symptom rating				
YMRS, total, M (SD)	2.51 (3.57)			
MADRS, total, M (SD)	7.39 (8.95)			
BPRS, total, M (SD)	33.24 (9.32)			
BPRS, positive psychotic subscale, M (SD)	4.63 (1.64)			
CGI-BP, mania severity, M (SD)	1.15 (.573)			
CGI-BP, depression severity, M (SD)	2.05 (1.563)			
CGI-BP, overall severity, M (SD)	2.02 (1.491)			

*FEM* first episode mania, *HC* healthy controls, *WTAR* Wechsler Test of Adult Reading, *YMRS* Young Mania Rating Scale, *MADRS* Montgomery–Åsberg Depression Rating Scale, *BPRS* Brief Psychiatric Rating Scale, *CGI-BP* Clinical Global Impression scale for bipolar illness, *t test* independent samples *t* test, *χ* ^*2*^ Chi-square test

^a^Missing data (FEM, *n* = 1; HC, *n* = 1)

^b^Diagnosis of bipolar I disorder confirmed by treating clinician (*n* = 1)

^c^Continuity correction

On average the FEM participants were in remission from acute mania (YMRS, *M* = 2.51, SD = 3.57), did not have positive psychotic symptomatology in the BPRS (*M* = 4.63, SD = 1.64), and were rated normal/not ill in mania severity on the CGI-BP (*M* = 1.15, SD = 0.573). However, the FEM participants were on average mildly depressed (MADRS, *M* = 7.39, SD = 8.95), as also identified in the BPRS total psychopathology rating (*M* = 33.24, SD = 9.32). FEM participants were considered minimally ill in depression severity (*M* = 2.05, SD = 1.56), and in overall bipolar disorder severity on the CGI-BP (*M* = 2.02, SD = 1.49).

### Cognitive functioning

The median and minimum/maximum scores for each group per cognitive measure and the associated test statistics and effect sizes are presented in Table 2.

**Table 2:** Cognitive functioning in participants following FEM relative to healthy controls

	FEM	HC	Test statistic	Value	*df*	*p* value	Effect size
	Median (min–max)	Median (min–max)					
Intelligence (WASI)^a^							
Verbal IQ	102 (63–128)	109 (96–130)	M–W	297.5	61	.084	0.22
Performance IQ	104 (75–128)	115 (90–129)	M–W	280	61	.046	0.26
Full-scale IQ	107 (70–128)	113 (98–126)	M–W	250.5	61	.014	0.31
Processing speed							
Trail Making Test-A^a^	31 (19–72)	22 (13–41)	M–W	120.5	61	< .001	0.57
Digit symbol coding^a^	73 (30–106)	80 (60–125)	M–W	207	61	.002	0.4
Go/No-Go^b^, go reaction time	294.96 (238.13–424.11)	304.91 (258.77–387.96)	M–W	355	60	.480	0.09
Cogstate^c^, detection reaction time	2.44 (2.32–2.69)	2.43 (2.36–2.59)	M–W	362	58	.668	0.06
Attention							
Digit Span forward^a^	8 (4–12)	10 (5–12)	M–W	370	61	.532	0.08
Go/No-Go^b^, go misses	0 (0–8)	0 (0–6)	M–W	382	60	.753	0.04
Stroop^d^, congruent errors	2.5 (0–15)	2 (0–13)	M–W	374.5	58	.928	0.01
Cogstate^c^, identification reaction time	2.66 (2.55–2.91)	2.66 (2.54–2.80)	M–W	345.5	58	.487	0.09
Sustained attention^e^							
ANT, alerting reaction time	44.61 (− 99.46 to 162.3)	38.56 (− 21.45 to 97.28)	M–W	373	59	.785	0.04
ANT, orienting reaction time	164.15 (13.67–316.42)	159.54 (15.70–232.76)	M–W	364	59	.677	0.05
ANT, executive control reaction time	226.74 (6.37–794.91)	189.81 (84.25–273.07)	M–W	281	59	.081	0.23
ANT, no cue errors	1 (0–5)	1 (0–3)	M–W	358	59	.593	0.07
ANT, spatial cue errors	1 (0–5)	1 (0–2)	M–W	335.5	59	.351	0.12
ANT, double cue errors	1 (0–5)	1 (0–10)	M–W	378.5	59	.850	0.02
ANT, congruent errors	0 (0–2)	0 (0–3)	M–W	329.5	59	.248	0.15
ANT, incongruent errors	3 (0–9)	2.5 (0–13)	M–W	341.5	59	.432	0.1
Verbal learning and memory^a^							
Rey auditory verbal learning test^e^—trial 1	6 (3–12)	7.5 (4–12)	M–W	215	61	.002	0.39
Rey auditory verbal learning test^e^—trials 1–5	52 (26–66)	59.50 (42–71)	M–W	170.5	61	< .001	0.47
Rey auditory verbal learning test^e^—delayed recall	11 (3–15)	14 (10–15)	M–W	174	61	< .001	0.47
Non-verbal learning and memory							
Cogstate, one-card learning^f^	1.01 (0.26–1.22)	1.03 (0.82–1.12)	M–W	339.5	57	.609	0.07
Cogstate, Groton Maze Learning Test, delayed, errors^g^	6 (0–24)	4 (0–11)	M–W	291.5	57	.187	0.17
Working memory							
Digit Span backward^a^	4 (1–10)	7 (3–12)	M–W	186.5	61	.001	0.44
Verbal fluency^a^							
FAS	37 (17–59)	43 (17–58)	M–W	309.5	61	.122	0.2
Animal category	21 (11–32)	23 (16–27)	M–W	280	61	.045	0.26
Executive functions							
Trail Making Test-B^h^	75 (31–215)	51 (30–116)	M–W	207	60	.004	0.37
Go/No-Go^b^, no-go false alarms	9.5 (2–21)	6.5 (1–16)	M–W	282	60	.063	0.24
Stroop effect^d^, reaction time	360.02 (97.20–603.79)	380.58 (115.75–618.27)	M–W	364	58	.794	0.03
Stroop^d^, incongruent errors	7 (1–26)	6 (3–24)	M–W	378	58	.974	< 0.01
Cogstate^c^, Groton Maze Learning Test, errors	54 (23.00–142.00)	51.00 (32.00–115.00)	M–W	347	58	.502	0.09

*FEM* first episode mania, *HC* healthy controls, *ANT* attention network test, *M–W* Mann–Whitney *U* test

ANT, alerting reaction time = no cue reaction time − double cue reaction time

ANT, orienting reaction time = double cue reaction time − spatial cue reaction time

ANT, executive control reaction time = incongruent trials reaction time − congruent trials reaction time

ANT, no cue errors = total errors for the no cue conditions

ANT, spatial cue errors = total errors for the spatial cue conditions

ANT, double cue errors = total errors for the double cue conditions

ANT, congruent errors = total errors for the congruent trials

ANT, incongruent errors = total errors for the incongruent trials

Stroop effect = incongruent condition reaction time − congruent condition reaction time

Stroop, congruent errors = total errors for the congruent trials

Stroop, incongruent errors = total errors for the incongruent trials

Go/No-Go, go reaction time = reaction time for go responses

Go/No-Go, no-go false alarms = sum of false responses to no-go stimulus

Go/No-Go, go misses = sum of missed responses to go stimulus

^a^Missing data (HC, *n* = 1)

^b^Missing data (FEM, *n* = 1; HC, *n* = 1)

^c^Missing data (FEM, *n* = 5)

^d^Missing data (FEM, *n* = 3; HC, *n* = 1)

^e^Missing data (FEM, *n* = 2; HC, *n* = 1)

^f^Missing data (FEM, *n* = 4; HC, *n* = 1)

^g^Missing data (FEM, *n* = 5; HC, *n* = 1)

^h^Missing data (HC, *n* = 2)

#### Intelligence

FEM participants had significantly lower FSIQ (*p* = .014) and PIQ (*p* = .046) than HCs, with medium (*r* = .31) and small to medium effect (*r* = .26), respectively. However, PIQ did not remain significant after family-wise adjustment (i.e. *α*/3). There was no significant difference between groups in VIQ (*p* = .084).

#### Processing speed

A highly significant group difference was observed between groups on the TMT-A (*p* < .001) and digit symbol coding (*p* = .002), even after family-wise adjustment (i.e. *α*/4). FEM patients performed substantially slower than HCs with a large (*r* = .57) and medium (*r* = .40) effect size, respectively.

There were no significant differences between groups in ‘go’ reaction time (*p* = .480) or Cogstate^TM^ Detection time (*p* = .668).

#### Attention

The groups did not differ significantly on Digit Span-forward (*p* = .532), missed go responses (*p* = .753), Stroop congruent total errors (*p* = .928) or in Cogstate_TM_ Identification time (*p* = .487).

#### Sustained attention

FEM and HC groups performed similarly in ANT alerting (*p* = .785), orienting (*p* = .677) and executive control (*p* = .081). There was no significant difference between groups in total errors for the no cue (*p* = .593), spatial cue (*p* = .351), double cue (*p* = .850), congruent (*p* = .248) and incongruent (*p* = .432) conditions.

#### Verbal learning and memory

FEM patients recalled significantly fewer words in trial 1 of the RAVLT relative to HCs (*p* = .002), of medium effect (*r* = .39). FEM patients recalled significantly fewer words than HCs in trials 1–5 (*p* < .001) and in delayed verbal recall (*p* < .001), which were both of a medium to large effect size (*r* = .47). These differences remained significant after family-wise adjustment (i.e. *α*/3).

#### Non-verbal learning and memory

There were no significant group differences in the OCL task (*p* = .609) or in the GMLT-delayed recall (*p* = .187).

#### Working memory

FEM patients had poorer working memory capacity than HCs, with a highly significant difference between groups in Digit Span-backward (*p* = .001) with medium to large effect (*r* = .44).

#### Verbal fluency

There was no significant difference between groups in phonemic fluency (*p* = .122). FEM participants produced significantly fewer words than HCs in semantic fluency (*p* = .045), though, this difference did not remain significant after family-wise adjustment (i.e. *α*/2).

#### Executive functions

A large difference was observed in cognitive flexibility with FEM patients performing worse than HCs in TMT-B (*p* = .004), even after family-wise adjustment (i.e. *α*/5), with a medium effect size (*r* = .37).

Groups did not differ significantly with respect to response inhibition in Go/No-Go false alarm responses (*p* = .063), in Stroop incongruent total errors (*p* = .974), or in Stroop effect (*p* = .794). Additionally, no significant group differences were observed in spatial problem solving as assessed by GMLT (*p* = .502).

### Relationship between clinical symptomatology and cognition

A significant negative correlation was found between rating scores on the YMRS and the RAVLT trial 1 (*p* = .033) with a moderate effect size (*r* = − .333). There also was a significant negative relationship between symptom rating on the YMRS and scores on the RAVLT trials 1–5 (*p* = .029), which was of a moderate effect size (*r* = − .342). Furthermore, a significant negative correlation was found between general psychopathology rating (BPRS total) and the RAVLT trials 1–5 (*p* = .008), which was of a moderate to large effect size (*r* = − .408). There were no other significant correlations found between clinical symptom rating scales and cognition.

## Discussion

The purpose of this study was to investigate cognitive functioning in youth following FEM. FEM patients were found to have lower FSIQ than HCs. While the groups were statistically matched in estimated premorbid intelligence, the difference in premorbid IQ was of medium effect and so the difference in current FSIQ may be driven by true pre-existing group differences or the inherent difficulties encountered matching FEM and HC on premorbid intelligence. Furthermore, FEM patients were still within the average intelligence range when compared to norms of people from a similar age group. Furthermore, the groups did not significantly differ in verbal and performance IQ after controlling for multiple comparisons. Previous research has been mixed showing that patients following FEM had poorer global intellectual functioning compared to HCs (Elshahawi et al. 2011; López-Jaramillo et al. 2010) and that FEM patients and HCs did not differ in verbal IQ, though FEM patients performed more poorly than HCs in subtests of performance IQ, including Block Design (Hellvin et al. 2012) and spatial reasoning (Hellvin et al. 2012; Torres et al. 2010).

Our findings indicate that FEM patients displayed cognitive impairments in some, but not all areas of cognitive functioning. First, as expected FEM patients performed worse than HCs in tests of processing speed, verbal learning and memory, working memory and cognitive flexibility. Second, phonemic verbal fluency and non-verbal learning and memory were not impaired relative to HCs; and differences observed in sematic verbal fluency were no longer apparent after family-wise adjustment. Contrary to our hypothesis, there were no group differences found in attention span, sustained attention or in the computerised tests of psychomotor speed and response inhibition. Although findings in the literature examining cognitive functioning in the early stages of bipolar disorder is somewhat mixed (Daglas et al. 2015), the results from the current study are generally comparable to the majority of research findings in FEM.

Regarding processing speed, Elshahawi et al. (2011) also revealed impairments in FEM patients relative to HCs as assessed by TMT-A and Digit Symbol Coding. Additionally, Hellvin et al. (2012) found that FEM patients performed worse than HCs in Digit Symbol Coding, whilst reporting no difference between groups in Stroop performance. On the other hand, Torres et al. (2010) found that FEM patients and HCs performed alike in both Stroop and in TMT-A. Another study by López-Jaramillo et al. (2010) reported a similar performance between FEM patients and HCs in TMT-A and in Digit Symbol Coding; however, this study had recruited relatives of the FEM patients as HCs, which may have influenced their findings. It has been reported that first-degree relatives of people with bipolar I disorder have a slower ability to process information than those without a family history of psychiatric illnesses (Antila et al. 2007).

Deficits in working memory have been reported by most first episode studies across the bipolar disorder spectrum relative to HCs (Barrett et al. 2009; Elshahawi et al. 2011; Hellvin et al. 2012; Hill et al. 2009; López-Jaramillo et al. 2010). However, two studies that assessed working memory using the letter–number sequencing (LNS) task failed to find a significant difference between groups (Torres et al. 2010; Zanelli et al. 2010). An explanation for this may be that FEM patients may have a weaker activation of the phonological loop required for verbal memory encoding (as in the Digit Span task), but may have maintained the ability to process more complex information, such as the interaction required between visuospatial functions, processing speed, and working memory when switching between letters and numbers in LNS (Crowe 2000; Haut et al. 2000).

Our study revealed that FEM patients had deficits in verbal learning and memory, but spared non-verbal learning and memory. Notably, when either mania or general psychopathology symptoms increased, verbal learning and memory performance decreased. Similar to our findings, Torres et al. (2010) reported that patients who had recently experienced a first episode of mania recalled significantly fewer words than HCs. Whilst, another study failed to find a significant difference between groups in total words recalled (trials 1–5), there were a significantly higher percentage of FEM patients (24%) who had clinically impaired verbal learning on the task (1.5 standard deviations below the mean of the HC group) than HCs (5%) (Hellvin et al. 2012). Impairments in delayed verbal recall have also been reported by previous studies in people with bipolar disorder experiencing their first episode of mania or psychosis (Hellvin et al. 2012; Zanelli et al. 2010).

Most research on first episode patients across the bipolar spectrum has revealed that non-verbal learning and memory remains intact (Hellvin et al. 2012; López-Jaramillo et al. 2010; Torres et al. 2010; Zanelli et al. 2010). Only one study of patients with bipolar disorder in remission from their first episode of psychosis identified a deficit in non-verbal memory (Barrett et al. 2009). Furthermore, a study on individuals at ultra-high risk for psychosis identified impaired visual reproduction relative to HCs, in patients who later developed a first episode of psychosis (Brewer et al. 2005). A deficit in non-verbal memory has also been identified in patients with recurrent bipolar disorder (Arts et al. 2008). Thus, dysfunction in non-verbal memory may reflect a deficit primarily related to psychosis or more chronic forms of bipolar disorder that appears to remain unaffected following FEM.

Our finding that attention was not impaired is consistent with the majority of first episode studies in bipolar disorder (Hellvin et al. 2012; Hill et al. 2009; López-Jaramillo et al. 2010; Torres et al. 2010). Although one study identified impairment in attention span in FEM patients relative to HCs, this study was limited by their recruitment of hospital employees from the same hospital as the FEM patients for the HC group, and therefore may not have been truly representative of the general population (Elshahawi et al. 2011).

Furthermore, our finding of no deficit in sustained attention is contrary to a previous study by Torres et al. (2010), who found that FEM patients performed significantly worse in this domain than HCs. The inconsistency between these study findings may be largely attributed to the different tests that were administered. The sustained attention task used in the current study required the unique activation of alerting, orienting and executive control pathways, which is a variation from more commonly used tests of sustained attention such as the rapid visual information processing task. However, as there has only been one previous study that has assessed sustained attention in FEM, further studies are required in respect to this domain.

In accordance with the recent systematic review on FEM (Daglas et al. 2015), both phonemic and semantic verbal fluency remained intact in the current study of FEM patients. Whilst a recent meta-analysis in the first episode bipolar disorder identified deficits in verbal fluency, these were of small effect and were observed in only two studies (Lee et al. 2014). However, these studies had compared adult FEM patients to a poorly matched HC group in age, sex and/or education level (Nehra et al. 2006; Zanelli et al. 2010).

Furthermore, we identified that there were no impairments in most domains of executive function, except for cognitive flexibility. Cognitive inflexibility has been reported in the acute state of FEM (Fleck et al. 2008). Most studies on patients following FEM have reported that cognitive flexibility remained intact relative to HCs (Hellvin et al. 2012; López-Jaramillo et al. 2010; Torres et al. 2010), with the exception of Elshahawi et al. (2011) who identified deficits in cognitive flexibility during remission in patients who had FEM with psychotic features relative to HCs. Most studies in FEM during the acute state (Lebowitz et al. 2001) and in remission (López-Jaramillo et al. 2010; Torres et al. 2010) are in support of our finding that response inhibition is not impaired following FEM. The deficits in response inhibition identified by one study of FEM patients who were predominantly depressed at the time of testing may have been reflective of the ongoing mood symptoms (Hellvin et al. 2012). A study by Malhi et al. (2007) identified that depressed patients with bipolar disorder had significantly poorer response inhibition than HCs, a finding that was not observed for either the euthymic or hypomanic groups.

The decline in cognitive functioning with illness progression has been elucidated by studies comparing FEM patients to those with multiple episodes (Elshahawi et al. 2011; Hellvin et al. 2012; López-Jaramillo et al. 2010; Torres et al. 2010). Relative to findings of widespread cognitive impairments in people with chronic forms of bipolar disorder, our findings suggest that specific deficits in processing speed, verbal learning and memory, working memory and cognitive flexibility might occur from the early stages of the illness. It may be postulated that the impairments reported in attention, sustained attention, non-verbal memory, verbal fluency and other executive functions may result from recurrent episodes (Bora et al. 2009; Bourne et al. 2013; Mann-Wrobel et al. 2011; Torres et al. 2007), as reported deficits in these domains are largely inconsistent in studies in FEM (Daglas et al. 2015). Neuroimaging studies have provided evidence in support of neuroprogression in bipolar disorder, with findings of prefrontal, cerebellum volume and ventricular abnormalities seen in patients with recurrent episodes relative to first episode patients or HCs (DelBello et al. 1999; Mills et al. 2005; Strakowski et al. 2002). Some structural brain abnormalities pertaining to the subgenual prefrontal cortex, have also been identified early in the course of the illness, which may reflect the cognitive deficits in the specific domains observed by studies in FEM (Strakowski et al. 2005).

Amongst the strengths of this study is the relatively large sample size of a specified group of psychiatric patients recruited from a naturalistic treatment environment. Given the sample size, post hoc power analyses indicated that we had sufficient power at .80 to detect moderate to large effects when *α* was set at .05. Additionally, in this study we administered an extensive cognitive battery, which covered several cognitive domains and included computerised cognitive testing to increase sensitivity in identifying deficits. Importantly, the FEM participants were matched as closely as possible to the HC group in age and sex. We also attempted to match the groups as closely as possible on premorbid intelligence; however, the FEM group had an average premorbid intelligence score 6.8 lower than the HC. Although the between-group difference in premorbid intelligence was not statistically significant, it was of moderate effect and might have explained the differences between groups in specific cognitive domains. It was not possible to control for premorbid intelligence and other factors (e.g. education) in non-parametric analyses. However, it is argued, that clinically such a difference is not necessarily meaningful and both groups had mean premorbid intelligence scores that would be considered in the average or normal range. Also highlighted are the difficulties matching patient and healthy control groups on such variables.

Other limitations included that stabilisation from acute mania was based on clinical judgment without use of an objective mania cut-off score, and on average the FEM participants were mildly depressed. Medication effects may have influenced cognitive functioning; it would be methodologically ideal but ethically questionable to include a medication-naive group. Although this study utilised a catchment area service, the generalisability of our results is limited to individuals who had stabilised from a FEM with psychotic features, representing the more severe end of the bipolar spectrum. The findings of this study are also not generalisable to people following stabilisation from first episode mania on other medications. Additionally, this study did not exclude FEM participants with comorbidities such as substance abuse disorders, which may have impacted the findings. Also, due to the cross-sectional nature of this study, it is not possible to assess whether cognitive deficits existed prior to FEM.

## Conclusions

Our findings revealed lower global intelligence in people following FEM may be evident prior to the onset of FEM, as well as specific cognitive deficits in processing speed, verbal learning and memory, working memory, and cognitive flexibility. These findings highlight the necessity of cognitive testing early in the course of the disorder. Amid the clinically relevant findings of this study, the differences observed in verbal learning and memory compared to non-verbal learning and memory may inform tailored interventions to address potential difficulties in functioning in people with FEM. Future research on the trajectory of cognitive functioning following FEM and the associated effects of treatment medications over time is warranted.
